# Activation-induced cytidine deaminase in tertiary lymphoid structures: dual roles and implications in cancer prognosis

**DOI:** 10.3389/fonc.2025.1555491

**Published:** 2025-04-09

**Authors:** Zhuangwei Lv, Junna Jiao, Wuyang Xue, Xiaoyu Shi, Ruihan Wang, Jinhua Wu

**Affiliations:** ^1^ School of Forensic Medicine, Xinxiang Medical University, Xinxiang, China; ^2^ School of Basic Medical Sciences, Xinxiang Medical University, Xinxiang, China; ^3^ Xinxiang Engineering Technology Research Center of Immune Checkpoint Drug for Liver-Intestinal Tumors, Xinxiang Medical University, Xinxiang, China; ^4^ Department of Laboratory Medicine, Second Affiliated Hospital of Xinxiang Medical University, Xinxiang, China; ^5^ School of Junji College, Xinxiang Medical University, Xinxiang, Henan, China

**Keywords:** activation-induced cytidine deaminase (AID), germinal center reaction, TLSs, TIL-B cells, solid tumors

## Abstract

Activation-induced cytidine deaminase (AID) serves as a critical molecular orchestrator in the germinal center (GC) reaction within secondary lymphoid organs (SLOs), driving the production of high-affinity antibodies through somatic hypermutation. While its pathological implications are well-documented - including ectopic expression in non-B cell populations and transcriptional dysregulation linked to hematological malignancies and solid tumorigenesis - the cellular provenance of AID in solid tumors remains an unresolved paradox. This review advances two principal hypotheses: (1) AID may derive from tertiary lymphoid structures (TLSs), ectopic immune niches mirroring SLO organization, and (2) exhibits context-dependent transcriptional duality, capable of both potentiating and suppressing gene expression based on microenvironmental cues. Through systematic analysis of AID/GC involvement across cancer subtypes, we delineate mechanistic connections between lymphoid neogenesis and tumor progression. Our examination extends to TLS architecture, revealing three critical dimensions: (i) structural organization and cellular heterogeneity, (ii) developmental trajectories, and (iii) bidirectional interactions with tumor microenvironments. Crucially, we establish functional parallels between tumor-infiltrating B cells (TIL-Bs) in SLOs versus TLSs, while elucidating the differential roles of AID in canonical GC versus TLS-associated GC formation. This synthesis ultimately proposes that AID’s functional dichotomy - acting as both oncogenic collaborator and tumor suppressor - underlies the paradoxical prognostic associations observed with TLS presence across malignancies. The review thereby provides a conceptual framework reconciling AID’s dual functionality with the context-dependent immunobiology of tumor-associated lymphoid structures.

## 1 Introduction

Activation-induced cytidine deaminase (AID) drives antibody diversity and affinity maturation through somatic hypermutation (SHM) and class switch recombination (CSR) within germinal centers (GCs), thereby promoting B cell development and humoral immunity (1–15). However, dysregulated AID expression induces genotoxic off-target effects through aberrant SHM/CSR activity, a key driver of oncogenic mutations (16). Notably, tumor-associated AID expression demonstrates strong spatial correlation with tumor-infiltrating B cells (TIL-Bs). These lymphocytes are predominantly localized within tertiary lymphoid structures (TLSs), ectopic immune aggregates that recapitulate lymphoid organogenesis in tumor microenvironments (17–21).

In this review, we initially address the dual biological role of AID: it can suppress tumor progression by silencing oncogenic pathways (e.g., through DNA hypermethylation) or promote malignancy by inducing mutagenesis via off-target deamination (16, 22, 23). Next, we present a summary of the research findings regarding AID’s involvement in the formation of GCs within secondary lymphoid structures (SLOs) and its association with cancers (16, 24, 25). Moreover, we provide an in-depth analysis of the formation of tertiary lymphoid structures (TLS) and the resulting two-sided impact on tumor prognosis (26–30). Finally, we discuss the interrelationship between AID and TLS, emphasizing AID-expressing tumor-infiltrating B cells (TIL-Bs) and TLS-associated GCs (TLS-GCs), which serve as functional hubs within tumor microenvironments (31, 32). We propose that AID’s functional duality in TLS-GCs–mediated by its mutagenic and immunomodulatory activities–underlies the paradoxical prognostic effects of TLSs across cancer types.

## 2 The biochemistry and physiological functions of AID

AID (Activation-Induced Cytidine Deaminase) was originally identified as a novel gene product in murine CH12F3-2 B lymphoma cells, where it regulates immunoglobulin diversification. As a member of the APOBEC (apolipoprotein B mRNA editing enzyme, catalytic polypeptide-like) family, AID harbors a conserved cytidine deaminase domain responsible for its catalytic activity (33). Structurally, AID is characterized as a cytidine deaminase containing an APOBEC1-homologous domain, enabling its enzymatic function to mediate cytidine-to-uridine (C→U) conversions in nucleic acids (34, 35). While APOBEC family members primarily target RNA substrates, AID exhibits unique biochemical specificity as a single-stranded (ss) DNA-selective cytidine deaminase. This enzymatic activity catalyzes the deamination of deoxycytidines (dCs) to deoxyuridines (dUs) in DNA molecules, distinguishing it from canonical APOBEC proteins (1, 2). This dual functional capacity - evolutionary conservation of RNA-editing motifs coupled with ssDNA substrate preference - underpins AID’s specialized roles in genomic remodeling.

The human *AICDA* gene, located on chromosome 12 (12p13.31), encodes the 198-amino acid activation-induced cytidine deaminase (AID), a DNA-editing enzyme critical for adaptive immunity. AID’s functional architecture comprises two evolutionarily conserved domains: 1) a cytidine deaminase catalytic core that converts cytosine (C) to uracil (U) in DNA, generating U:G mismatches, and 2) an APOBEC1-homologous domain enabling genome-wide recognition of the WRCY motif (W = A/T, R = A/G, Y = C/T). This dual-domain synergy allows AID to selectively target ssDNA regions, induce C→U deamination at defined sequence contexts, and thereby initiate two essential processes for immunoglobulin diversification—somatic hypermutation (SHM) to enhance antibody affinity through targeted point mutations, and class switch recombination (CSR) to alter antibody effector functions via DNA recombination (1, 3, 4, 6, 8–10).

### 2.1 AID and SHM, CSR

AID serves as the central driver of somatic hypermutation (SHM) and class switch recombination (CSR) through its sequence-specific recognition of the WRCY motif (“hotspot”). During SHM, AID generates U:G mismatches via cytosine deamination in immunoglobulin heavy (IgH) and light (IgL) chain variable region exons. These mismatches are processed through DNA replication, base excision repair (BER), or mismatch repair (MMR), ultimately producing C→A/T/G substitutions and insertions/deletions that diversify antibody affinity (1–10, 13–15, 36, 37). In CSR, AID induces double-strand breaks (DSBs) in switch regions (Cμ/Cγ/Cα/Cδ/Cϵ) of the IgH constant region, which encode distinct antibody isotypes (IgM/IgG/IgA/IgD/IgE). Antigen stimulation triggers IgH transcription, forming stable R-loop DNA: RNA hybrids that expose single-stranded DNA (ssDNA) in switch (S) regions. For instance, during IgM-to-IgG switching, AID targets ssDNA in Sμ and Sγ regions, creating U:G mismatches that are converted to DSBs via BER/MMR. Subsequent repair mediates VDJ-Cγ1 recombination through Sγ1-Sμ looping, enabling precise deletion of intervening constant regions while retaining antigen specificity in the expressed IgG1 heavy chain (Igγ1) (1–10, 38–43).

### 2.2 AID’s epigenetically modification function

#### 2.2.1 AID and DNA demethylation

Beyond its canonical roles in somatic hypermutation (SHM) and class switch recombination (CSR), AID exhibits an evolutionarily conserved capacity to initiate active DNA demethylation through 5-methylcytosine (5mC) processing. AID mediates this epigenetic regulation by deaminating 5mC to thymine, triggering subsequent base excision repair (BER) and mismatch repair (MMR) pathways that ultimately replace methylated cytosines with unmodified bases, thereby promoting genome-wide demethylation (44). While the relative contributions of BER versus MMR in methyl group removal remain debated, accumulating evidence positions AID as a key facilitator of dynamic DNA demethylation through this mechanism (4, 15, 45). This paradigm is strongly supported by zebrafish embryo studies demonstrating a two-step demethylation process: 1) AID-catalyzed 5mC deamination generates T:G mismatches, followed by 2) BER-mediated replacement through species-specific glycosylases–methyl-CpG binding domain protein 4 (MBD4) in zebrafish and thymine-DNA glycosylase (TDG) in mammals (46–48).

AID catalyzes the deamination of 5-hydroxymethylcytosine (5hmC), a product generated by ten-eleven translocation (TET) family enzymes through 5-methylcytosine (5mC) hydroxylation, into 5-hydroxymethyluracil (5hmU) (49, 50). The base excision repair (BER) system, involving MBD4 or TDG, subsequently replaces 5hmC with unmodified cytosine (46–48). Emerging evidence reveals a bidirectional regulatory relationship between AID and TET family members, particularly TET2, through transcriptional and post-transcriptional mechanisms. These interactions coordinate AID-associated active demethylation processes in cancer biology, with significant implications for hematological malignancies (51, 52). Notably, AID facilitates TET2-mediated demethylation of the FANCA gene as a transcriptional cofactor in diffuse large B-cell lymphoma (DLBCL) (22), while paradoxically collaborating with DNMT1 to maintain methylation at the BCL6 promoter (23). Beyond gene-specific epigenetic regulation, AID deficiency induces genome-wide methylation alterations, suggesting its dual role in both promoting and suppressing DNA methylation through mechanisms extending beyond its canonical enzymatic activity. This systemic impact positions AID as a global modulator of epigenetic landscapes rather than merely executing localized methylation changes.

#### 2.2.2 AID in cellular reprogramming

Cellular reprogramming, the conversion of somatic cells (e.g., fibroblasts) into pluripotent stem cells, has been successfully achieved through overexpression of defined transcription factors including OCT4, SOX2, KLF4, and c-MYC to generate induced pluripotent stem cells (iPSCs) (53, 54). This revolutionary approach offers therapeutic potential for diverse pathologies including various cancers, cardiovascular diseases, metabolic disorders, and chronic inflammatory conditions. Emerging evidence suggests AID’s DNA demethylation activity may facilitate pluripotency acquisition through interactions with gene clusters in oocytes and primordial germ cells (PGCs) (55, 56). Experimental models using mouse-human interspecies heterokaryons (generated by fusing embryonic stem cells with fibroblasts) demonstrated that OCT4 (POU5F1) and NANOG reactivation during reprogramming requires AID-mediated promoter demethylation - AID depletion impairs these processes by preventing activation of methylated OCT4 and NANOG promoters in somatic cells (53, 56). These findings establish AID’s essential role in mammalian cellular reprogramming through active DNA demethylation, as corroborated by multiple experimental systems (4, 15, 49, 50, 53–56). *In vivo* investigations using germinal center B cells (GCBs) from wild-type versus AID-deficient (Aicda-/-) mice revealed altered patterns of differentially methylated cytosines (DMCs) during naive B cell-to-GCB differentiation, indicating AID-dependent epigenetic remodeling during physiological cell state transitions (57). Given its dual capacity to enable artificial reprogramming and regulate natural differentiation processes, AID emerges as a promising therapeutic target for neoplastic and systemic disorders, highlighting its potential to modulate cellular plasticity through epigenetic mechanisms.

### 2.3 New faces: AID’s emerging role in gene transcription

While AID was originally characterized for its canonical role in immunoglobulin gene diversification through cytidine deamination-mediated somatic hypermutation (SHM) and class switch recombination (CSR), emerging evidence reveals multifaceted contributions to carcinogenesis and transcriptional regulation. Although AID’s off-target activity at non-Ig loci has been implicated in approximately 8% of cancer cases (58), recent studies demonstrate that its non-enzymatic functions in transcriptional modulation may constitute the predominant oncogenic mechanism (22, 23). The molecular machinery enabling AID’s genomic targeting shows striking parallels between Ig and non-Ig contexts: In antibody gene diversification, AID requires collaboration with Spt5, RPA, and RNA PolII to access single-stranded DNA substrates that facilitate double-strand breaks (DSBs) (35–37, 59–63). Similarly, in diffuse large B-cell lymphoma (DLBCL), AID exhibits dual transcriptional regulation - cooperating with TET2 to activate FANCA expression while partnering with DNMT1 to repress BCL6 (22, 23). These observations establish two critical principles: (1) AID functions as a context-dependent transcriptional cofactor, and (2) its regulatory outcomes (activation vs. repression) depend on interacting partners within distinct molecular complexes. This mechanistic plasticity enables AID to exert bidirectional effects on gene expression, translating to either tumor-promoting or tumor-suppressive roles depending on cellular context. Such functional duality challenges conventional paradigms and underscores the need for system-specific evaluation of AID’s contributions to oncogenesis.

However, The characterization of AID as a functional cofactor remains controversial, as over 50 reported binding partners are widely considered likely experimental artifacts rather than biologically relevant interactions (64). This skepticism arises from AID’s intrinsic physicochemical properties as a highly charged, structurally dynamic molecule prone to nonspecific electrostatic associations with diverse proteins under experimental conditions (65, 66). While these promiscuous binding tendencies complicate the validation of genuine functional partnerships, emerging evidence suggests a subset of these interactions may reflect context-dependent biological activities. This paradox underscores the critical need for stringent validation through orthogonal methodologies to distinguish physiologically meaningful associations from spurious interactions inherent to AID’s biochemical nature.

### 2.4 Regulation of AID expression and implications in disease

AID, initially recognized for its role in antibody diversification in B cells, exhibits broader implications in non-B cells, where its dysregulation may contribute to carcinogenesis (67). Chronic inflammation, a key cancer risk factor, promotes aberrant AID expression via TNFα-NF-κB signaling in inflammation-driven cancers like *H. pylori*-associated gastric cancer and colitis-related colon cancer (68, 69). Ectopic AID expression in tumors correlates with mutations in oncogenes such as *TP53*, highlighting its mutagenic potential (70).

AID forms a self-reinforcing loop by partnering with Gadd45 to activate Pax5, which in turn binds the AID promoter, amplifying its own expression. This auto-activation mechanism perpetuates off-target effects, including oncogene promoter mutations and chromosomal translocations (71).

As a transcriptional cofactor, AID interacts with RNA Polymerase II and SPT5 to influence transcription elongation. While its highly charged structure allows nonspecific protein binding, functional partnerships (e.g., with SPT5) appear critical for targeting active transcription sites (35). Paradoxically, AID can both activate and repress gene expression, acting as a molecular “balancer” that fine-tunes oncogene activity. Though its precise regulatory mechanisms remain elusive, emerging evidence underscores AID’s dualistic nature—mediating both genomic instability and epigenetic regulation—in cancer biology.

## 3 AID’s role in GC formation during carcinogenesis

### 3.1 Germinal center

#### 3.1.1 Anatomy and cellular composition of GC

**Figure 1:**
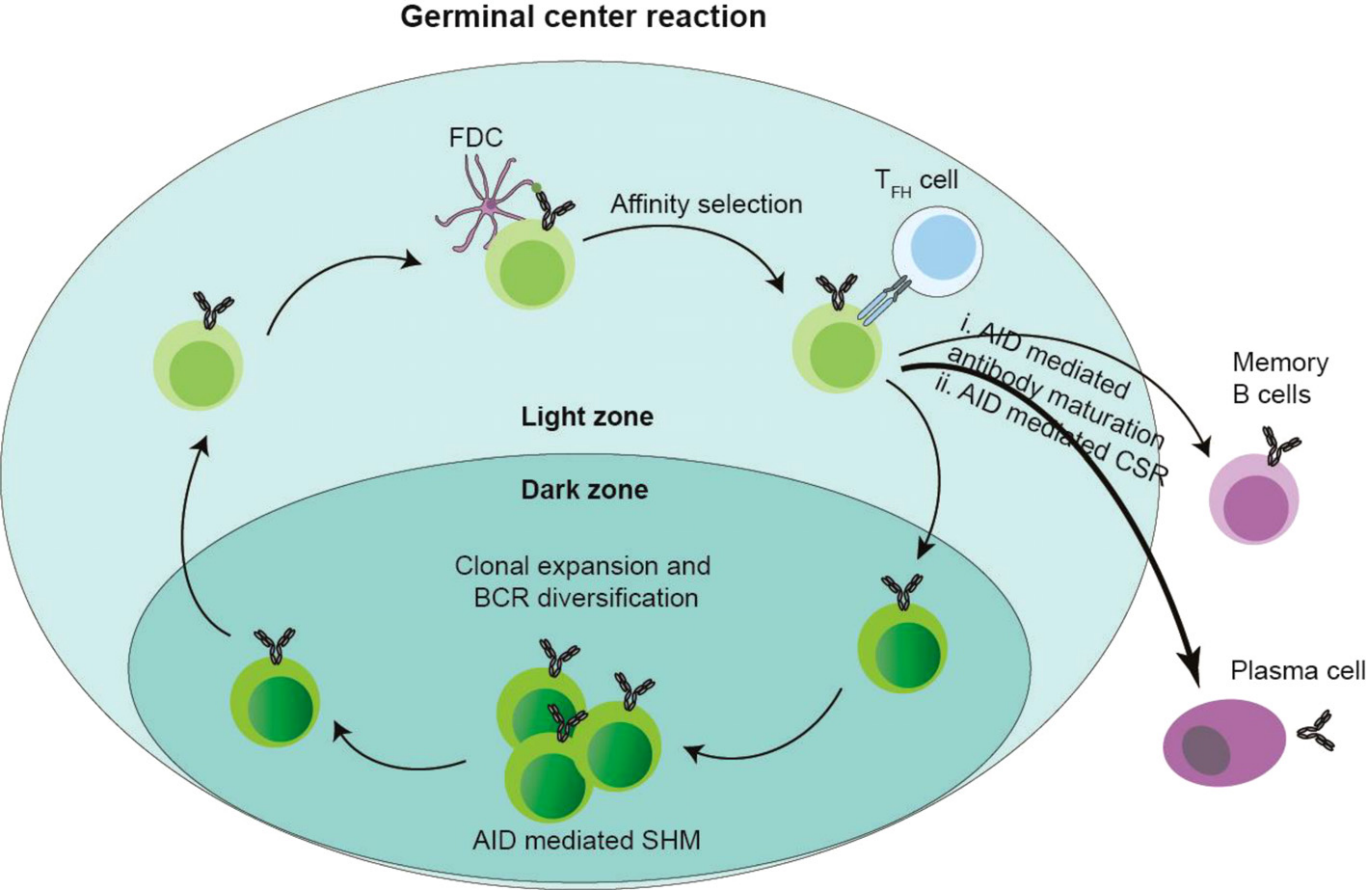
Germinal center (GC) and AID. After antigen stimulation, the GC structure formed by a series of regulation. The follicular marginal zone B cells around GC capture antigens, and these antigens were presented by follicular dendritic cells (FDCs) to CD4^+^ Follicular T helper cells (T_FH_), T_FH_ induces AID-mediated SHM of GC B cells in the GC dark zone and CSR of GC B cells in the GC light zone. Then the B cells in the GC light zone are selected depending on the antigen-antibody affinity presented by FDCs and T_FH_ cells, and the B cells with low antibody affinity undergo apoptosis, or re-enter the dark and bright areas of GC to cause a new round of SHM and CSR; while the B cells with high-affinity antibodies migrate out of GC, differentiate into plasma cells and memory B cells, and are reactivated when they are re-challenged by antigens.

Naive B cells, having completed V(D)J recombination of immunoglobulin heavy (IgH) and light (IgL) chain genes via RAG1/RAG2-mediated recombination in the bone marrow, migrate to peripheral lymphoid tissues where they localize to IgM^+^IgD^+^ interfollicular regions adjacent to T cell zones (44, 55). Antigen presentation in subcapsular sinuses facilitates coordinated B-T cell interactions, with T zone-derived signals promoting full B cell activation and T follicular helper (TFH) cell differentiation (72, 73). Activated B cells subsequently relocate to follicular centers, where follicular dendritic cell (FDC)-supported proliferation generates expanding B cell clusters that displace resident follicular B cells, establishing early germinal centers (GCs) (74, 75). Histologically distinct GC subcompartments emerge: the dark zone contains densely packed centroblasts undergoing AID-driven somatic hypermutation (SHM) of Ig variable regions, while the light zone features FDC networks and TFH cells that mediate affinity-based selection (76–78). Following SHM in the dark zone, B cells migrate to the light zone where FDC-presented antigens test antibody affinity. Low-affinity clones either undergo apoptosis or re-enter the SHM/class switch recombination (CSR) cycle, while high-affinity variants exit the GC to differentiate into plasma cells or memory B cells capable of rapid antigen recall responses (**Figure 1**) (79). This cyclical process of mutation and selection optimizes antibody diversity and specificity within the dynamic GC microenvironment.

#### 3.1.2 Molecular control of GC reaction

Germinal center (GC) B cells exhibit fundamental biological distinctions from their naive B cell precursors. First, while naive B cells achieve functional maturity through sequential V(D)J recombination of immunoglobulin heavy (IgH) and light (IgL) chains during development, forming complete B cell receptors (BCRs), GC B cells undergo secondary diversification via AID-mediated somatic hypermutation (SHM) of variable regions and class switch recombination (CSR) in constant regions, enhancing antibody diversity and isotype flexibility (44). Second, GC B cells display remarkable proliferative capacity with cell cycles lasting 5-6 hours, contrasting sharply with the quiescent state of naive B cells - a metabolic transformation reflected in their enlarged cellular morphology and heightened biosynthetic activity (80–83). Third, GC B cells represent transitional precursors that ultimately differentiate into memory B cells and antibody-secreting plasma cells, completing the terminal phases of B cell development (84, 85). These specialized properties necessitate precise molecular regulation of GC reactions, governed by coordinated gene networks (including AID, BCL6, MYC, IRF4, IRF8, and BLIMP1) that balance proliferative expansion with genomic instability from DNA breakage and repair processes (86, 87). The interplay of these mechanisms enables the GC microenvironment to sustain rapid clonal expansion while facilitating affinity maturation and antibody diversification.

**Figure 2:**
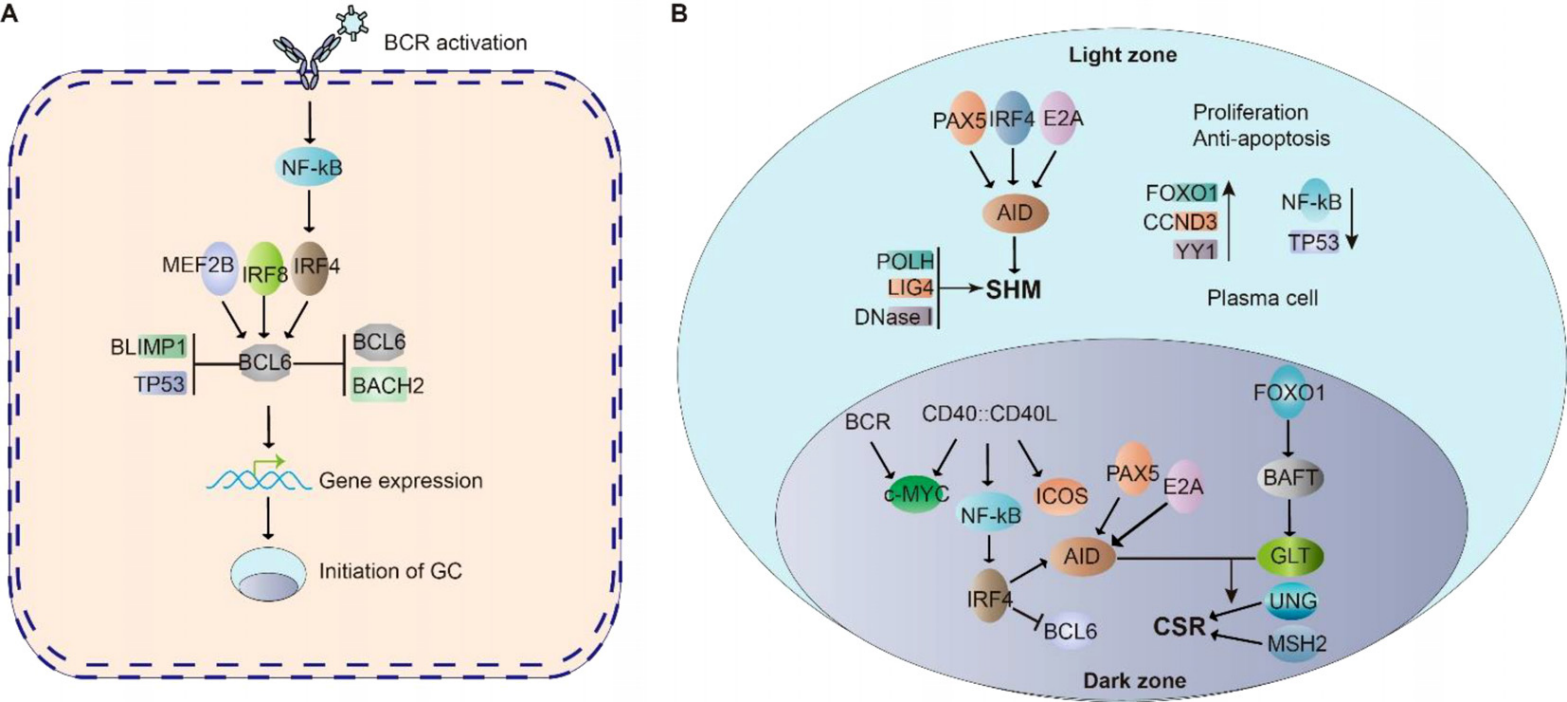
Cell signaling in Germinal center (GC). **(A)** BCR signaling activate NF-κB, which initiate BCL6 expression. BCL6 is essential for the initiation of germinal center. MEF2B, IRF8, IRF4, BLIMP1 and TP53 regulate BCL6 expression. BCL6 and BACH2 repress BCL6 expression. The cooperation of these signaling allow the initiation of GC. **(B)** In the GC dark zone, AID is a critical enzyme for SHM; PAX5, E2A, and IRF8 control AID expression. POLH, LIG4 and DNaseI highly expressed in DZ B cells to promote SHM. FOXO1 is responsible for maintaining the GC dark zone B cell program, CCND3, YY1, NF-κB and c-MYC are critical for GC dark zone B cell proliferation and survival. In the GC light zone, CD40-CD40L signaling stimulated NF-κB further activates IRF4 expression, which suppresses BCL6 gene. PAX5, E2A, and IRF4 are key factors in regulating the expression of AID. FOXO1 motivates its downstream target BATF, regulates germline transcripts. GLT levels are key factors to elevate the accessibility of AID to gene body in CSR.

Following antigen stimulation, B cell receptor (BCR) signaling activates downstream transcriptional regulators including the NF-κB pathway, octamer-binding transcription factors OCT1/OCT2, and their coactivator OBF1 (OCA-B/Bob1) (88–90). These factors orchestrate germinal center (GC) formation through coordinated regulation of BCL6, the master transcriptional regulator of GC biology. In CD4+ T cells, OBF1 and OCT1/OCT2 directly bind the BCL6 promoter to initiate its transcription, establishing a reciprocal regulatory loop: BCL6 promotes T follicular helper (TFH) cell differentiation, while TFH-B cell interactions further amplify BCL6 expression (91–93). This self-reinforcing mechanism stabilizes GC architecture through BCL6-mediated upregulation of CXCR4, which regulates dark zone B cell positioning and GC compartmentalization (94). Concurrently, interferon regulatory factors (IRFs) exhibit temporal control over GC dynamics. Early-phase IRF4, activated by BCR signaling in outer follicular B cells, collaborates with IRF8 to initiate BCL6 transcription during GC initiation (94–96). As the reaction progresses, IRF4 transitions to a repressive role, downregulating BCL6 to facilitate GC resolution. This dual-phase regulation by IRF4 ensures precise control of GC lifespan, while IRF8 maintains early transcriptional activation (**Figure 2A**). The integration of these pathways highlights the multilayered regulation of GC formation, balancing sustained proliferation with eventual termination through dynamic transcriptional networks.

Following germinal center (GC) formation, the spatial organization of the dark zone (DZ) and light zone (LZ) is defined by the differential expression of chemokine receptors CXCR4 and CXCR5, respectively (97). Within this microenvironment, activation-induced cytidine deaminase (AID) plays a critical role in facilitating somatic hypermutation (SHM) and class-switch recombination (CSR)–two essential processes for antibody diversification. In GC dark zone B cells, transcriptional regulators including PAX5, E2A, and IRF4 coordinately upregulate AID expression to drive these genomic alterations (4, 24). This process is further supported by the predominant expression of SHM-associated DNA repair machinery components (POLH, LIG4, and DNase I) in dark zone B cells. FOXO1 emerges as a central coordinator of the dark zone program, maintaining AID protein stability through post-translational modifications to ensure efficient SHM (98). Furthermore, the transcription factor YY1 has been shown to exert dual regulatory functions in dark zone B cells, being indispensable for both cellular proliferation and survival through mechanisms involving metabolic reprogramming and anti-apoptotic signaling (99, 100) (**Figure 2B**).

Following rapid expansion in the germinal center (GC) dark zone, B cells migrate to the GC light zone where they undergo three critical processes: (i) antibody affinity maturation to produce high-affinity antibodies, (ii) class switch recombination (CSR), and (iii) differentiation into either plasma cells or memory B cells (101). In the light zone microenvironment, CD40 signaling activates NF-κB to enhance IRF4 expression, which subsequently suppresses BCL6 transcription (102, 103). The activation-induced cytidine deaminase (AID) enzyme remains upregulated through cooperative regulation by PAX5, E2A, and IRF4 (4, 24). Facilitated by follicular dendritic cells (FDCs), AID mediates both CSR and somatic hypermutation during affinity maturation (1–10).The CSR process is further regulated by BATF, a FOXO1 downstream target that controls germline transcript (GLT) expression in centrocytes (104). GLT levels directly correlate with chromatin accessibility for AID-mediated DNA modifications. Concurrently, IRF4 collaborates with BLIMP1 to drive plasma cell differentiation through dual mechanisms: suppressing GC B cell proliferation while activating plasma cell differentiation programs (94, 95, 105). This differentiation pathway is opposed by BCL6, which maintains GC B cell identity by repressing PRDM1 (encoding BLIMP1) and STAT5 transcription (106).The cell fate decision is further modulated by lineage-specific regulators. Activated B cell Factor-1 (ABF-1), predominantly expressed in memory B cells, promotes memory cell differentiation from centrocytes (107). This intricate regulatory network ensures proper balancing between antibody-producing plasma cells and long-lived memory B cells (**Figure 2B**).

However, the molecular regulation of GC reaction involves a plethora of molecules orchestrated by a complex gene network. The exact underlying molecular mechanism remains unclear, and the regulation of gene networks in the context of the GC reaction is largely uncharted territory.

### 3.2 AID associated GC in hematological malignancies

The germinal center (GC) reaction begins with antigenic challenge of naive B cells, initiating a differentiation cascade that produces centrocytes, plasmablasts, and ultimately either long-lived plasma cells or memory B cells. This developmental progression crucially depends on activation-induced cytidine deaminase (AID)-mediated somatic hypermutation (SHM) and class switch recombination (CSR), which drive antibody diversification and functional specialization. Notably, dysregulation at any differentiation node–particularly during AID-dependent genomic remodeling –creates susceptibility to malignant transformation. Such developmental aberrations are clinically significant as they frequently underlie hematological malignancies, with approximately 90% of lymphomas originating from B cell lineage defects during GC maturation (5, 108–113).

Approximately 80% of germinal center (GC)-derived B cell lymphomas are categorized as B cell non-Hodgkin lymphomas (B-NHLs), encompassing three major subtypes: Burkitt lymphoma (BL), follicular lymphoma (FL), and diffuse large B cell lymphoma (DLBCL) (114–116). Anatomically distinct GC microenvironments contribute to lymphoma pathogenesis - BL predominantly originates from dark zone B cells undergoing rapid proliferation, while FL typically develops from light zone centrocytes engaged in affinity maturation (117). DLBCL demonstrates broader developmental plasticity, potentially arising from dysregulated B cells at any GC transitional stage. This spectrum includes two molecular subtypes: 1) germinal center B cell-like (GCB) DLBCL, which emerges during active GC development, and 2) activated B cell-like (ABC) DLBCL, associated with differentiation arrest at the plasmablast-to-plasma cell transition during early post-GC maturation (118).

The pathogenesis of hematological malignancies—particularly B-cell lymphomas arising from dysregulated germinal center (GC) reactions—is strongly associated with diverse genetic abnormalities, including point mutations and recurrent chromosomal translocations (13). Notably, GC-derived B-cell non-Hodgkin lymphomas (B-NHLs) frequently exhibit translocation events mechanistically linked to activation-induced cytidine deaminase (AID) activity. In sporadic Burkitt lymphoma, erroneous AID-mediated class-switch recombination (CSR) drives immunoglobulin locus (Ig)-MYC translocations, while diffuse large B-cell lymphomas (DLBCLs) commonly harbor Ig-BCL6 translocations through similar mechanisms. Furthermore, AID-dependent somatic hypermutation (SHM) processes contribute to additional translocation patterns observed in these malignancies. These genomic aberrations collectively promote lymphomagenesis by disrupting oncogene regulation, enhancing genomic instability, and conferring survival advantages to malignant clones (109–111).

While essential for germinal center (GC) establishment and function, activation-induced cytidine deaminase (AID) exhibits a paradoxical role in lymphomagenesis through its enzymatic activity. The enzyme’s physiological capacity to induce targeted DNA breaks–facilitating somatic hypermutation and class switching–becomes oncogenic when combined with defective DNA repair mechanisms, particularly error-prone non-homologous end joining pathways (30). This genomic instability manifests clinically as point mutations, chromosomal translocations (e.g., MYC-IgH), and clonal evolution. Strikingly, AID-deficient murine models demonstrate ~80% reduction in chromosomal translocations and oncogenic mutations compared to wild-type counterparts (119). These mechanistic insights confirm AID’s dual functionality: its indispensable role in adaptive immunity is counterbalanced by intrinsic genotoxic risks that contribute significantly to B cell lymphomagenesis, highlighting its pathological significance in GC-derived malignancies.

### 3.3 AID associated GC in solid tumors

Under physiological conditions, activation-induced cytidine deaminase (AID) specifically targets immunoglobulin (Ig) genes to mediate somatic hypermutation (SHM) and class switch recombination (CSR) during germinal center reactions. However, pathological contexts reveal AID’s capacity for promiscuous genomic targeting, termed AID’s off-target activity, which extends to oncogenes (e.g., BCL6), tumor suppressors (e.g., TP53), and genomic stability regulators (e.g., ATR) (16). This aberrant activity manifests through three distinct oncogenic mechanisms:Genomic Instability: Off-target deamination induces mutagenesis and facilitates recurrent chromosomal translocations (e.g., c-MYC/IgH), particularly when coupled with error-prone DNA repair pathways. Ectopic AID expression in non-lymphoid cells further exacerbates genomic instability, contributing to both hematological and epithelial malignancies (16). Epithelial Plasticity: Aberrant AID activity in epithelial tissues promotes epithelial-mesenchymal transition (EMT) through transcriptional reprogramming, facilitating tumor invasion and metastasis (16, 25). Epigenetic Remodeling: AID’s recently characterized role in active DNA demethylation enables cellular reprogramming via TET-mediated oxidation pathways. This epigenetic regulatory capacity presents novel therapeutic opportunities for cancer treatment through differentiation therapy (4, 15, 51–55).

While tumor-infiltrating T lymphocytes (TIL-Ts) are well-established immune components in tumors (120–122), B cells also extensively infiltrate solid malignancies, forming tumor-infiltrating B-cell subsets (TIL-B) that predominantly organize into tertiary lymphoid structures (TLSs) or occasionally exist as isolated clusters (17–21). Recent studies by Silina et al. have classified TLS organization in lung tumors into three distinct stages: early TLS (E-TLS), characterized by mixed CD3^+^ T-cell and CD20^+^ B-cell infiltrates; primary follicle-like TLS (PFL-TLS), featuring CD21^+^ follicular dendritic cell (FDC) networks that stabilize lymphoid architecture; and secondary follicle-like TLS (SFL-TLS), containing germinal center-like aggregates (GC-TLSs) marked by CD23+ TIL-associated germinal centers (TIL-GCs) (26, 123, 124). These GC-TLSs, identifiable through co-expression of BCL6, Ki67, and activation-induced cytidine deaminase (AID), recapitulate physiological germinal center reactions encompassing initiation, maturation, and maintenance phases (27, 28). Notably, AID emerges as a critical regulator in tumor-associated TLS development, with its functional interplay in GC-TLSs underscoring its therapeutic potential as a target for amplifying antitumor immune responses through TLS modulation (27, 28).

## 4 Tertiary lymphoid structures

Physiologically, secondary lymphoid organs (SLOs) such as lymph nodes develop to regulate antibody diversity and facilitate affinity maturation. Tertiary lymphoid structures (TLSs) demonstrate remarkable morphological, cellular, and molecular similarities to SLOs (26). These ectopic lymphoid aggregates are frequently observed in pathological contexts including chronic inflammatory sites, autoimmune disorders, and tumor microenvironments (29, 30). The architectural organization of mature TLSs mirrors that of SLOs, featuring distinct compartmentalization: a central B cell follicle containing naive B cells, surrounded by a germinal center (GC) with proliferating B cells, and an adjacent T cell zone populated by T lymphocytes and dendritic cells (DCs) (**Figure 2**) (26). Notably, key chemokines involved in SLO development - including CCL19, CCL21, CXCL13, and CXCL12 - are consistently detected in TLSs (125). Furthermore, well-established TLSs contain stromal cell populations resembling fibroblastic reticular cells (FRCs) and follicular dendritic cells (FDCs), which are essential stromal components of SLOs (29, 30). This structural and molecular conservation between SLOs and TLSs strongly suggests that shared organizational mechanisms may govern TLS formation. However, the precise role of these conserved elements as potential TLS organizers requires further experimental validation through focused mechanistic studies.

### 4.1 Inducers and organizers of TLS formation

TLSs are ectopic structures that share similarities with SLOs. They are characterized by the aggregation of immune cells, including B cells, T cells, dendritic cells, high endothelial venules (HEV), and fibroblasts (126).

In the developmental organization of secondary lymphoid organs (SLOs), the coordinated interaction between lymphoid tissue inducer (LTi) cells and lymphoid tissue organizer (LTo) cells is essential (127, 128). Within lymph nodes, LTi cells—a subset of innate lymphoid cells—collaborate with mesenchymal-derived LTo cells, which subsequently differentiate into stromal components such as follicular dendritic cells (FDCs) and fibroblastic reticular cells (FRCs) (127, 128). In contrast, the cellular orchestrators of ectopic tertiary lymphoid structure (TLS) formation in humans remain poorly defined. However, mechanistic insights from murine tumor models with TLSs suggest functional parallels: T and B cells expressing lipoteichoic acid (LTA) have been identified as potential LTi-like cells, while podoplanin-positive (PDPN+) fibroblasts expressing lymphotoxin-beta receptor (LTBR) may act as LTo-like cells (128). This cellular interplay drives TLS organization, with activated PDPN+ fibroblasts upregulating key chemokines such as CXCL13 and CCL19/CCL21. These chemotactic signals recruit immune cells expressing corresponding receptors (CXCR5 and CCR7, respectively), thereby facilitating the spatial organization and maintenance of TLS microarchitecture (29).

### 4.2 Cellular composition of TLS

**Figure 3:**
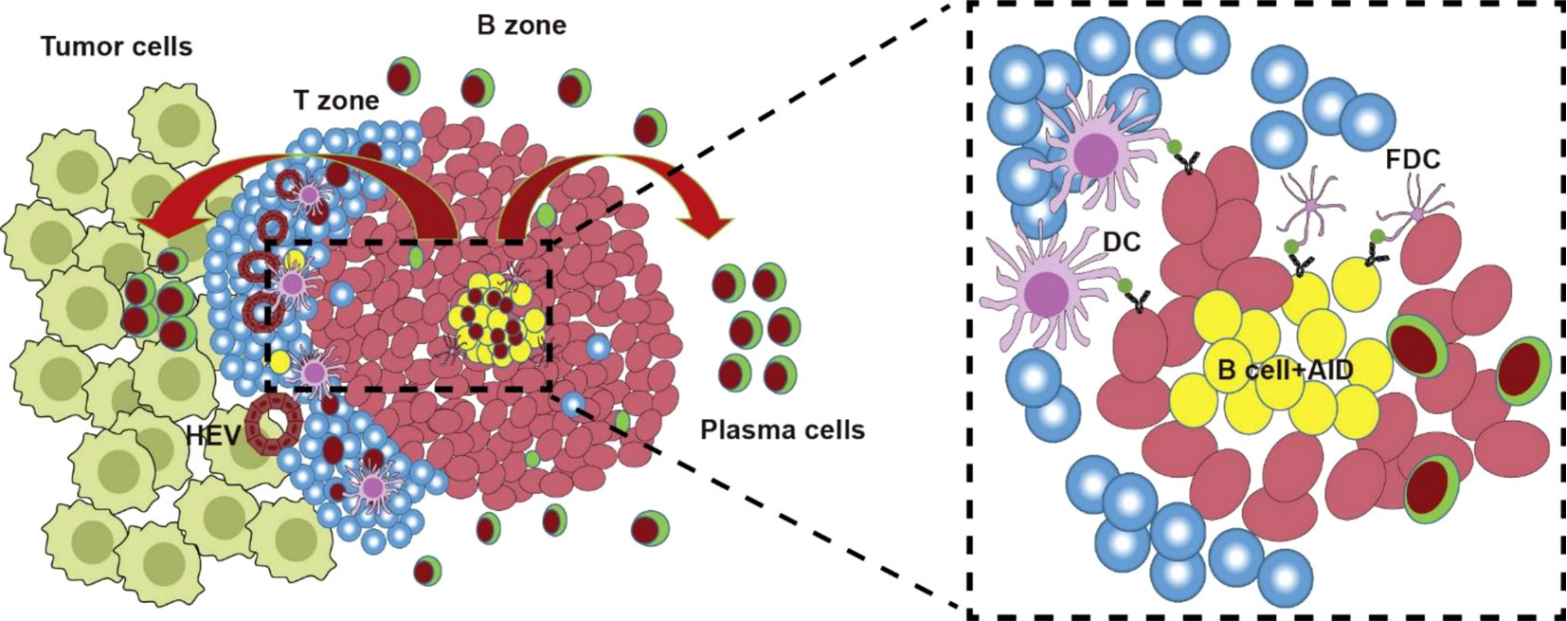
TLS and AID. TLS forms during tumor formation. The TLS was similar to GC structure containing T zone and B zone. After antigen presentation by FDCs to T_FH_, T_FH_ helps AID-mediated AID-driven SHM and CSR of Ig genes for antigen-driven affinity maturation in GC reaction. Then B cells with low antibody affinity undergo apoptosis, or re-enter a new round of SHM and CSR; while B cells with high-affinity antibodies migrate out of GC, differentiate into plasma cells and memory B cells.

Histopathological characterization using hematoxylin and eosin (H&E) staining and immunohistochemical markers (e.g., CD20, CD3, CD4, CD8, PNAd, and DC-LAMP) has consistently demonstrated that tertiary lymphoid structures (TLSs) comprise organized aggregates of innate and adaptive immune cells encircled by high endothelial venules (HEVs) (129). In malignancies such as melanoma and non-small cell lung cancer (NSCLC), tumor-associated TLSs exhibit architectural and cellular parallels to secondary lymphoid organs (SLOs), containing distinct zones of T lymphocytes (CD3+), mature dendritic cells (DC-LAMP+), and follicular B cells (CD20+) (130). The maturation spectrum of TLSs ranges from loosely aggregated immune cell clusters (indicative of immature/early TLSs) to highly organized structures featuring compartmentalized follicles with germinal centers (GCs) and tumor antigen-specific T/B lymphocytes, mirroring the functional organization of SLOs (**Figure 3**) (131).Recent advances in multiplex immunophenotyping have refined the understanding of TLS cellular dynamics. Proliferative GC B cells (Ki67+CD23+) within TLSs express activation-induced cytidine deaminase (AID)—critical for somatic hypermutation (SHM) and class-switch recombination (CSR)—along with Bcl6, the master transcriptional regulator of GC B cell differentiation (26, 29, 31). Stromal networks of follicular dendritic cells (FDCs), identified by CD21 or CD23 expression, underpin TLS-GC microarchitecture. The peri-GC regions are populated by macrophages (CD68+), cytotoxic CD8+ T cells, CD4+ T helper subsets, and specialized follicular helper T cells (Tfh; Bcl6+PD-1+ICOS+IL-21+). Terminally differentiated plasma cells (CD38+CD138+) frequently localize to TLS peripheries, collectively indicating coordinated humoral immunity (antibody production) and cytotoxic effector functions within mature TLSs (29, 31, 117, 120).

### 4.3 TLS presence and composition in tumors

The tumor microenvironment (TME) plays a pivotal role in tumor initiation, progression, and metastasis through dynamic interactions between malignant cells and their surrounding milieu (132, 133). This complex ecosystem comprises not only the structural, functional, and metabolic characteristics of tumor tissues but also extends to the intracellular environment of tumor cells, including nuclear and cytoplasmic components (132, 133). Tumor cells actively manipulate their microenvironment through autocrine and paracrine signaling mechanisms, creating self-sustaining conditions that favor their proliferation and survival (134). Conversely, systemic and local modifications in metabolic pathways, secretory profiles, immune responses, and tissue architecture exert regulatory influences that can either promote or constrain tumor development (132–135). Within this intricate network of interactions, tumor-associated tertiary lymphoid structures (TLSs) have emerged as critical functional components of the TME, demonstrating significant biological relevance in tumor immunology (29).

TLSs have been observed not only in autoimmune diseases and chronic inflammation but also, significantly, in various types of cancers. In the context of cancer, TLSs serve as specialized microenvironments for cancer-specific antigen presentation and antibody production. However, it is important to note that different types of cancer may exhibit distinct subsets of TLS cells (28). While specific cells and chemokines are believed to play a role in orchestrating TLS formation within tumors, the exact mechanisms underlying this process remain unclear.

### 4.4 Bilateral impact of TLS on tumor prognosis

TLSs are frequently observed in the context of chronic infection, where they promote localized antiviral immune responses via the activation of naive T cells. However, in the case of tumor-associated TLSs, they often serve as a dual prognostic indicator for patient survival (32). Numerous studies have highlighted the importance of the densities of high endothelial venules (HEVs), CD4^+^ T cells, particularly TFH subsets, B cells, and mature DCs in defining TLSs. Additionally, there appears to be a positive correlation between TLS density, levels of intratumoral T and B cells, and a Th1/cytotoxic immune profile among tumor-infiltrating lymphocytes (TILs) (26, 28–30).

Emerging evidence indicates that tumor-associated tertiary lymphoid structures (TLSs) may function as critical hubs for initiating antitumor immunity by activating newly infiltrated naïve T and B cells (129–131). Alternatively, their presence might reflect preexisting robust intratumoral CD8+ T cell effector activity—a well-established biomarker for favorable cancer prognosis. Notably, TLSs in triple-negative breast cancer (TNBC), Merkel cell carcinoma, and gastric tumors correlate with improved clinical outcomes, marked by organized immune architectures containing moderate tumor-infiltrating lymphocytes (TILs), Th17 cells, T-bet^+^ cells, and CD20^+^ B cells (**Figure 3**) (136, 137). Paradoxically, however, certain malignancies exhibit adverse associations with TLSs. In colorectal cancer (CRC), TLS presence coincides with advanced disease stages, while in breast cancer, it correlates with higher tumor grades, diminished intratumoral immune cell infiltration, and increased lymph node metastasis (26). Murine lung adenocarcinoma models further reveal TLSs as recruitment sites for immunosuppressive regulatory T cells (Tregs), a mechanism similarly observed in human lung transplant studies (138, 139). These dualistic roles—enhancing antitumor responses in some contexts while fostering immunosuppression in others—underscore TLSs’ potential as precision therapeutic targets for modulating cancer immunity (140, 141).

Taken together, these studies suggest that the prognostic value of TLSs is dependent on the type of tumor. It is possible that different types of tumors elicit qualitatively distinct immune responses, which can be distinguished by their effector and regulatory mechanisms. These responses may also evolve over time. In some tumors or at certain stages, these immune cells may primarily derive from conventional tumor-draining secondary lymphoid organs (SLOs), while in other cases, they may arise from tumor-associated TLSs (26–28). This highlights the notion that the “quality” of TLSs may confer, or result from, distinct types of immunity or immune regulation.

## 5 AID and TLS in tumors

### 5.1 TIL-B cells in tumors

The mechanisms driving tumorigenesis and malignant progression involve intricate, multistep biological processes. To improve cancer patient survival, significant research efforts have focused on developing targeted anti-tumor strategies. Among these, immunotherapy has emerged as a revolutionary frontier in oncology. Immuno-oncology research encompasses both the dynamic interplay between the tumor microenvironment (TME) and immune cells and the design of therapeutic interventions to augment existing cancer therapies. Notably, tumor-infiltrating immune cells are increasingly recognized as critical mediators of anti-tumor immunity, with their functional states and spatial distribution within the TME directly influencing therapeutic outcomes (142).

Tumor-infiltrating T cells (TIL-Ts) represent primary cellular targets for enhancing immunotherapy efficacy due to their central role in antitumor immunity (17, 18, 122). However, tumors harbor diverse immune populations, including functionally significant B-cell subsets collectively termed tumor-infiltrating B cells (TIL-Bs) (19–21). Emerging evidence underscores the therapeutic potential of TIL-B-targeted strategies, with studies over the past decade demonstrating their capacity to modulate antitumor responses. TIL-Bs comprise heterogeneous subpopulations—naïve B cells, germinal center (GC) B cells (TIL-GCs), memory B cells (TIL-Bmem), and antibody-secreting plasma cells (TIL-PCs)—each exhibiting distinct functional roles within the tumor microenvironment (TME) (28).

The critical role of B cells in secondary lymphoid organs (SLOs)—particularly in generating high-affinity plasma cells (PCs) and memory B cells (Bmem) during adaptive immune responses—is well-established. Beyond their classical roles in SLOs, tumor-infiltrating B cells (TIL-Bs) are increasingly recognized for their prognostic and therapeutic significance in cancer immunotherapy. For instance, TIL-B subpopulations, such as tumor-infiltrating plasma cells (TIL-PCs), have been linked to improved clinical outcomes in patients receiving immune checkpoint inhibitors (ICIs). In non-small cell lung cancer (NSCLC), elevated TIL-PC levels correlate with prolonged overall survival in anti-PD-L1-treated patients, a phenomenon associated with lymphoid aggregation and tertiary lymphoid structure (TLS) formation (143). Further supporting their functional relevance, NSCLC studies reveal that exhausted PD-1hi CD8+ T cells recruit B cells via CXCL13 secretion during anti-PD-1 therapy, as demonstrated by Thommen et al. (144). Collectively, these findings from human tumor analyses highlight TIL-Bs’capacity to orchestrate antitumor immunity, positioning them as pivotal contributors to immunotherapy efficacy.

Emerging evidence demonstrates that tumor-infiltrating B cells (TIL-Bs) exhibit broad antigen-targeting capabilities, engaging foreign antigens, self-antigens, and tumor-specific antigens within the tumor microenvironment (TME). The antibodies produced by tumor-infiltrating plasma cells (TIL-PCs) may originate from preexisting germline precursors or undergo affinity maturation in tertiary lymphoid structure-associated germinal centers (TLS-GCs). This antibody production is likely driven by the overexpression of immunogenic antigens in the TME (145). Functionally, TIL-PC-derived antibodies can mediate antitumor effects through multiple mechanisms: direct neutralization of tumor antigens, antibody-dependent cellular cytotoxicity (ADCC) via recruitment of natural killer (NK) cells, and opsonization-enhanced phagocytosis by macrophages and dendritic cells. Despite these advances, key questions remain unresolved, including the precise contribution of TIL-B-derived antibodies to tumor control, their specificity profiles across cancer types, and the regulatory pathways governing their functional plasticity in immunosuppressive TME niches. Further mechanistic studies are required to fully elucidate TIL-Bs’therapeutic potential and limitations in oncology.

### 5.2 AID and TLS-GC formation

Tertiary lymphoid structures (TLSs) are ectopic lymphoid aggregates observed in diverse pathological contexts, including infectious diseases, autoimmune disorders, cancers, and transplant rejection. Structurally analogous to secondary lymphoid organs (SLOs), TLSs harbor germinal centers (GCs) where B cells undergo proliferation, somatic hypermutation (SHM), and class-switch recombination (CSR) of immunoglobulin (Ig) genes to achieve antigen-driven affinity maturation (31, 32). Central to these processes is the enzyme activation-induced cytidine deaminase (AID), which catalyzes SHM and CSR in both SLO-derived GCs and TLS-associated GCs (TLS-GCs) (31, 32). Despite their ectopic nature, TLS-GCs recapitulate key functional and molecular features of SLO-GCs, including AID’s dual role in adaptive immunity and genomic instability. Within TLS-GCs, AID not only facilitates antibody diversification but also induces off-target effects, such as mutagenesis of non-Ig genes (e.g., oncogenes or tumor suppressors) and transcriptional regulation as a cofactor (16, 22, 23, 146). These off-target activities—linked to promoter mutations, chromosomal translocations, and dysregulation of cancer-related genes—underscore AID’s capacity to drive carcinogenesis. Conversely, AID-mediated transcriptional modulation can silence or activate oncogenic pathways, revealing its context-dependent regulatory duality (16, 22, 23, 146). This mechanistic ambivalence mirrors the paradoxical role of TLSs in tumor biology, where they may either foster antitumor immunity or promote malignant progression. The interplay between AID’s dual functions and TLS activity thus provides a molecular framework to explain their bidirectional impact on tumorigenesis (**Figure 3**).

### 5.3 AID and tumor-associated TLS

Ongoing studies are actively investigating the interplay between activation-induced cytidine deaminase (AID) and tumor-associated tertiary lymphoid structures (TLSs). TLSs are enriched with lymphocytes exhibiting immune activation markers, including proliferating (Ki67^+^) cells, AID-expressing B cells undergoing somatic hypermutation (SHM), and T cells polarized toward Th1/Tc1 differentiation (Tbet^+^), all of which correlate with favorable clinical outcomes (26–28, 31, 48, 130, 131). These observations suggest that the functional activity of TLS-resident lymphocytes—rather than TLS presence alone—may drive antitumor effects. Quantitative analyses further reveal dynamic compositional features within TLSs, such as the frequency of Ki67^+^ proliferating lymphocytes, AID^+^ B cells, and CD4^+^/CD8^+^ T cells with Th1/Tc1 phenotypes, which collectively reflect immune activation states (26–28, 31, 48, 130, 131). Notably, B cells in tumor-associated TLSs exhibit hallmark features of antigen-driven affinity maturation: AID-dependent antibody class switching, clonal expansion, and SHM. These processes are tightly regulated by local antigen presentation, facilitated by T follicular helper (Tfh) cell interactions within the TLS microenvironment (27, 72, 91). Collectively, these findings position AID not only as a mediator of B cell diversification but also as a molecular bridge linking TLS functionality to adaptive antitumor immunity. The spatial and temporal coordination of AID activity, antigen presentation, and lymphocyte activation within TLSs underscores their potential as immunological hubs capable of shaping tumor progression and therapeutic responses.

As critical components of the tumor microenvironment (TME), all B-cell subsets—from naïve B cells to antibody-secreting plasma cells (PCs)—exert multifaceted roles that are intrinsically linked to the composition and maturation of tumor-associated tertiary lymphoid structures (TLSs) (147, 148). Within the TME, tumor-infiltrating B cells (TIL-Bs) encounter diverse antigens, including foreign, self-, and tumor-specific antigens, which they internalize and process. In immature TLSs, B cells exhibit broad responsiveness to antigenic stimuli, marked by activation-induced cytidine deaminase (AID) expression that initiates early adaptive responses (18, 149). Antigen-driven AID activation further triggers a regulatory network promoting the development of TLS-associated germinal centers (TIL-GCs), a pivotal step in TLS maturation (31, 32, 145). Immature TLSs are characterized by immunosuppressive B-cell functions, where AID may facilitate low-affinity antibody production that inadvertently supports immune evasion. In contrast, mature TLSs enable B cells to undergo clonal expansion, AID-mediated somatic hypermutation (SHM), class-switch recombination (CSR), and affinity maturation, ultimately generating PCs that secrete high-affinity IgG or IgA antibodies (144, 145). Notably, TLS-derived PCs can produce antibodies targeting tumor-associated antigens; however, their functional outcomes depend on the immunological context of the TME. These antibodies may either mediate antitumor effects (e.g., via neutralization or antibody-dependent cytotoxicity) or paradoxically promote tumor progression (e.g., through immune complex-mediated inflammation or growth factor signaling) (150–152). This duality mirrors AID’s dual role in TLS biology—driving both protective immunity and oncogenic genomic instability—and underscores the context-dependent interplay between TLS maturation, antibody specificity, and tumor fate.

### 5.4 The impact of AID on the development of tertiary lymphoid structures

AID serves as a critical regulator in the formation and functional maturation of tertiary lymphoid structures (TLSs)–ectopic lymphoid aggregates that emerge under chronic inflammatory conditions or within tumor microenvironments. These organized lymphocyte clusters, composed of B and T cells in spatial arrangements resembling secondary lymphoid organs, have been clinically associated with enhanced anti-tumor immunity and improved prognostic outcomes in various cancers. AID’s principal biological function involves mediating two essential processes in B cell diversification: somatic hypermutation for antibody affinity optimization and class-switch recombination for antibody isotype modification. Emerging evidence indicates that AID activity extends beyond antibody diversification to support TLS development. In tumor-associated TLSs, upregulated AID expression facilitates B cell maturation and promotes the formation of germinal center-like microdomains, thereby enabling localized adaptive immune responses against malignancies (153, 154).The prognostic significance of TLSs in cancer progression has been closely linked to AID-driven immunomodulatory mechanisms. Through its enzymatic activity, AID enhances B cell receptor diversity within TLSs while simultaneously interacting with immune regulatory networks to maintain these lymphoid structures’ architectural integrity and functionality (155). This dual role positions AID as a molecular orchestrator that shapes the tumor immune microenvironment through both cell-intrinsic genetic modification and microenvironmental modulation. The synergistic relationship between AID and TLS components underscores the therapeutic potential of targeting this pathway to amplify anti-tumor immunity in chronically inflamed tissues.

### 5.5 AID-mediated mutations and tumorigenesis

AID plays a dual role in both physiological immunity and oncogenesis. While essential for generating antibody diversity through somatic hypermutation (SHM) and class-switch recombination (CSR) in normal B cells, AID’s mutagenic activity also drives genomic instability by introducing off-target mutations in non-immunoglobulin genes, including oncogenes and tumor suppressor loci. Within tertiary lymphoid structures (TLSs), this aberrant activity becomes particularly consequential. AID-mediated mutations in TLSs can accelerate tumor evolution by fostering genetic heterogeneity, thereby promoting aggressive cancer phenotypes. For example, in B-cell malignancies such as follicular lymphoma and chronic lymphocytic leukemia, AID-generated mutational signatures are recurrently linked to disease progression and therapy resistance (156, 157). These mutations often activate oncogenic pathways (e.g., MYC, BCL2) or disable tumor suppressors (e.g., TP53), directly enabling malignant transformation. Furthermore, TLSs create a permissive niche for AID dysregulation: the chronic inflammatory milieu and sustained antigen exposure in TLSs upregulate AID expression, amplifying its mutagenic impact and perpetuating a cycle of DNA damage and clonal selection (153, 158). Thus, AID’s role in TLSs epitomizes its paradoxical nature—orchestrating adaptive immunity while simultaneously fueling tumorigenic evolution.

### 5.6 The relationship between AID and immune responses

The interplay between AID and immune responses is multifaceted, particularly within tertiary lymphoid structures (TLSs). AID not only drives the production of high-affinity antibodies through somatic hypermutation and class-switch recombination but also supports B-cell differentiation into plasma cells, a cornerstone of adaptive humoral immunity. Within TLSs, AID’s influence extends beyond B-cell maturation: it shapes T-cell responses by modulating cytokine networks and fostering the differentiation of T follicular helper (Tfh) cells. These Tfh cells are indispensable for sustaining germinal center (GC) reactions, where they provide critical co-stimulatory signals to B cells, enabling robust antigen-specific antibody production (159, 160).Notably, TLSs enriched with AID activity correlate with enhanced responses to immunotherapy. By creating an immunologically active niche, TLSs promote T-cell infiltration, activation, and tumor-targeting efficacy, thereby improving checkpoint inhibitor outcomes. This synergy between AID-driven antibody diversification and T-cell priming underscores TLSs’role as hubs of coordinated antitumor immunity. Consequently, dissecting AID’s regulatory dynamics within TLSs offers dual insights: it elucidates mechanisms of immune evasion while revealing therapeutic strategies to amplify antitumor responses through selective modulation of AID activity, such as enhancing its protective roles or mitigating its oncogenic effects (19, 154).

## 6 Conclusion

The dual roles of AID and TLSs in tumor biology—coupled with AID’s critical function in germinal centers (GCs) and the GC-like architecture of TLSs—prompt an examination of AID’s contributions to TLS formation. Beyond its canonical role in antibody class-switch recombination (CSR) and somatic hypermutation (SHM), AID exerts context-dependent effects on tumor survival dynamics (161). First, AID exhibits paradoxical roles in tumor progression, either promoting oncogenesis through mutagenic activity or suppressing tumors via enhanced immune surveillance. Second, TLSs demonstrate dichotomous impacts on patient survival outcomes, with their prognostic value likely modulated by AID activity levels. Finally, AID facilitates the development of GC-like microanatomy within TLSs during tumorigenesis, directly linking its enzymatic function to TLS maturation and functionality (161). These interconnected roles position AID as a molecular linchpin bridging adaptive immunity, TLS biology, and tumor fate.

Emerging evidence demonstrates that AID engages in multifaceted transcriptional regulation through collaborative interactions with epigenetic modifiers such as DNMT1 and TET2, particularly modulating tumor-associated gene networks (22, 23). While AID is well-established as a genomic destabilizer in hematologic malignancies, driving pathogenic mutations and chromosomal translocations (129), recent studies reveal its capacity for dual regulatory functions extending beyond canonical deamination and demethylation activities (16, 22, 23). This functional duality manifests through context-dependent partnerships with transcriptional regulators, enabling AID to exert both positive and negative control over gene expression. Notably, AID facilitates the formation of germinal center (GC)-like tertiary lymphoid structures (TLSs) while simultaneously exhibiting tumor-modulating ambivalence–a paradoxical role that mirrors the observed dual effects of TLSs in tumor microenvironments, where immune activation and oncogenic progression may coexist depending on molecular coordination.

Our prior research has revealed non-canonical transcriptional regulatory functions of AID, yet its potential bidirectional regulatory effects on tertiary lymphoid structure (TLS) development and immune modulation within tumor microenvironments (TME) remain undefined. Urgent investigations are required to clarify how AID’s dualistic gene regulatory mechanisms–balancing transcriptional activation and suppression through dynamic partnerships with epigenetic modifiers –influence TLS formation and subsequent anti-tumor immunity. Given the proven clinical efficacy of engineered immune cell therapies, elucidating AID’s mechanistic involvement in TLS formation could uncover novel therapeutic targets for microenvironment-modulating anti-cancer strategies. While CAR-T cell therapy has demonstrated remarkable success in hematological malignancies (162), next-generation engineered immune effectors including CAR-NK cells and CAR-macrophages are emerging as promising therapeutic platforms (163). This understanding may inform the development of AID-enhanced CAR therapies that synergistically harness lymphoid neogenesis and immune cell engineering, translating mechanistic insights into clinical innovation.

## 7 Expert opinion

Emerging evidence suggests AID and tertiary lymphoid structures (TLSs) hold promise as biomarkers for immunotherapy responsiveness, yet critical questions must be resolved for clinical translation. A central challenge lies in determining whether AID differentially regulates immature versus mature TLS formation in tumors and how these distinct maturation states influence patient survival outcomes. This functional duality of AID–capable of both promoting lymphoid neogenesis and potentially driving tumor evolution through genomic instability – mirrors the paradoxical prognostic implications of TLSs in cancer progression. Therapeutic strategies aiming to amplify AID expression within tumor microenvironments (TME) could theoretically enhance TLS maturation, with proposed methodologies including localized delivery of AID-overexpressing vectors followed by immunohistochemical validation of TLS structural completeness. While mature TLSs may correlate with improved survival through enhanced antitumor immunity, their establishment might concurrently foster tumor heterogeneity via AID-mediated mutagenesis. These mechanistic investigations into AID’s spatiotemporal regulation of TLS development and functional polarization are essential for optimizing therapeutic interventions, particularly in designing combination strategies that maximize TLS-mediated immune activation while mitigating oncogenic risks inherent to AID activity.
